# The PTEX Pore Component EXP2 Is Important for Intrahepatic Development during the *Plasmodium* Liver Stage

**DOI:** 10.1128/mbio.03096-22

**Published:** 2022-11-29

**Authors:** Tahir Hussain, Jose Linera-Gonzalez, John M. Beck, Manuel A. Fierro, Gunnar R. Mair, Ryan C. Smith, Josh R. Beck

**Affiliations:** a Department of Biomedical Sciences, Iowa State University, Ames, Iowa, USA; b Roy J. Carver Department of Biochemistry, Biophysics and Molecular Biology, Iowa State University, Ames, Iowa, USA; c Department of Plant Pathology, Entomology and Microbiology, Iowa State University, Ames, Iowa, USA; NIAID/NIH

**Keywords:** EXP2, PTEX, *Plasmodium*, *glmS*, liver stage, malaria, nutrient transport, protein export, ribozyme

## Abstract

During vertebrate infection, obligate intracellular malaria parasites develop within a parasitophorous vacuole, which constitutes the interface between the parasite and its hepatocyte or erythrocyte host cells. To traverse this barrier, *Plasmodium* spp. utilize a dual-function pore formed by EXP2 for nutrient transport and, in the context of the PTEX translocon, effector protein export across the vacuole membrane. While critical to blood-stage survival, less is known about EXP2/PTEX function in the liver stage, although major differences in the export mechanism are suggested by absence of the PTEX unfoldase HSP101 in the intrahepatic vacuole. Here, we employed the glucosamine-activated *glmS* ribozyme to study the role of EXP2 during Plasmodium berghei liver-stage development in hepatoma cells. Insertion of the *glmS* sequence into the *exp2* 3′ untranslated region (UTR) enabled glucosamine-dependent depletion of EXP2 after hepatocyte invasion, allowing separation of EXP2 function during intrahepatic development from a recently reported role in hepatocyte invasion. Postinvasion EXP2 knockdown reduced parasite size and largely abolished expression of the mid- to late-liver-stage marker LISP2. As an orthogonal approach to monitor development, EXP2-*glmS* parasites and controls were engineered to express nanoluciferase. Activation of *glmS* after invasion substantially decreased luminescence in hepatoma monolayers and in culture supernatants at later time points corresponding to merosome detachment, which marks the culmination of liver-stage development. Collectively, our findings extend the utility of the *glmS* ribozyme to study protein function in the liver stage and reveal that EXP2 is important for intrahepatic parasite development, indicating that PTEX components also function at the hepatocyte-parasite interface.

## INTRODUCTION

Malaria remains one of the most devastating parasitic diseases in the world. In 2020, 241 million new infections occurred, resulting in 627,000 deaths, mostly among children under the age of 5 years in sub-Saharan Africa (1). Humans are infected by the bite of an anopheline mosquito that deposits sporozoites in the skin, which then travel to the liver and invade hepatocytes. A single round of development within this intrahepatic niche generates thousands of erythrocyte-infective forms, which are released into the circulation to initiate the pathogenic blood stage (2). As a critical bottleneck to patent *Plasmodium* infection in the mammalian host, the liver stage has emerged as an important drug and vaccine target.

Following invasion of both erythrocytes and hepatocytes, the parasite develops within a parasitophorous vacuole membrane (PVM) that forms the boundary of a compartment for expansive growth and the direct interface for host-parasite interactions (3, 4). In the blood stage, an arsenal of effector proteins is delivered across the PVM, remodeling the host cell to create a niche for intracellular development and avoid host defenses (5, 6). Protein export into the erythrocyte is facilitated by the *Plasmodium* translocon of exported proteins (PTEX) (7–9). The PVM-spanning channel of the translocon is formed by an oligomer of EXP2, the only widely conserved component of PTEX (10–12). EXP2 and its orthologs mediate small-molecule exchange between the host cytosol and PV lumen of vacuole-dwelling apicomplexans (13). However, *Plasmodium* spp. have additionally functionalized this nutrient-permeable channel through a flange-like adaptor formed by PTEX150 which docks the AAA+ chaperone unfoldase HSP101, transforming EXP2 into a protein-conducting PVM translocon (10, 11, 14). In this way, proteins destined for export are unfolded by HSP101 in the PV and passed through PTEX150 and EXP2 across the PVM and into the erythrocyte cytoplasm (11).

While small-molecule transport and protein export across the PVM are also anticipated to be critical for survival within the hepatocyte, the function of EXP2/PTEX is less clear during the liver stage. Intriguingly, while EXP2 and PTEX150 are also expressed during hepatocyte infection, HSP101 has not been detected until late liver-stage development, when it is loaded into the dense granules of forming merozoites to be deployed upon red blood cell (RBC) invasion (15–17). Furthermore, blood-stage exported proteins and reporters are not translocated beyond the liver-stage PVM, even when HSP101 is ectopically expressed at this stage, suggesting a distinct export mechanism (15, 18–21). Inactivation of *exp2* expression in sporozoites using the FLP/FRT stage-specific conditional knockdown system in Plasmodium berghei demonstrated a critical role for EXP2 in the transition from the mosquito host to the vertebrate blood stage (15). However, subsequent work surprisingly reported this to be the result of an invasion defect in EXP2-deficient sporozoites which could be rescued by exogenous supplementation with recombinant EXP2 or bacterial pore-forming toxins, implicating extracellular secretion of EXP2 in hepatocyte invasion through host membrane wounding (22).

While these findings imply a remarkable distinct function in hepatocyte entry, EXP2 is also expected to play an important role in PVM transport and possibly in effector protein export in the liver stage by analogy to its function in the blood stage (10, 14) as well as the role of the EXP2 orthologs GRA17 and -23 in PVM permeability in *Toxoplasma* (13). However, the functional importance of EXP2 during intrahepatic development remains unclear, as existing genetic strategies provide limited control of *exp2* knockdown timing in the liver stage. Here, we applied the *glmS* ribozyme to study the role of EXP2 during liver stage parasite development, showing for the first time that this system can be used to modulate parasite gene expression beyond the blood stage. This strategy enables temporal control of knockdown, revealing that EXP2 plays a critical role in intrahepatic development of liver-stage parasites independent of any contribution to invasion, consistent with a function in PVM nutrient uptake and/or protein export into the hepatocyte.

## RESULTS

### The *glmS* ribozyme enables inducible protein knockdown in the P. berghei blood and liver stages.

To study EXP2 function in *Plasmodium* liver-stage development after hepatocyte invasion, we sought to develop a ligand-based conditional knockdown approach that would enable more precise control of the timing of knockdown. We choose the *glmS* strategy, which involves introduction of a bacterial metabolite-responsive ribozyme sequence immediately downstream of the stop codon of the target gene (23–25). Knockdown is initiated by addition of glucosamine (GlcN) to the culture medium, which upon conversion to glucosamine-6-phosphate activates the ribozyme, resulting in cleavage of the mRNA 3′ untranslated region (UTR) and transcript destabilization, reducing target protein levels. The *glmS* ribozyme has been widely used to study protein function in the Plasmodium falciparum blood stage, where the ability to indefinitely culture the parasite *in vitro* allows GlcN to be delivered at a sufficient concentration and duration to mediate robust knockdown (25, 26). Indeed, the *glmS* approach is capable of producing lethal EXP2 knockdown in the P. falciparum blood stage (14). The *glmS* system has also been shown to function in the P. berghei blood stage, where ~2 mM GlcN was required to reduce the expression of a green fluorescent protein (GFP) reporter by 50% in culture, although difficulties in maintaining rodent malaria parasites *ex vivo* beyond a single developmental cycle limit the applicability of knockdown strategies, requiring the sustained presence of a ligand (27). Baseline GlcN serum concentrations are in the low nanomolar range in mice and can be increased only to ~2 μM by dietary supplementation (28), still far below the range observed to activate substantial *glmS* cleavage in *Plasmodium* (25, 27). Thus, while sufficient GlcN concentrations are unachievable for *in vivo* studies with *glmS*, endogenous GlcN levels are likely too low to significantly activate the ribozyme in the rodent host, so that tagging important or essential P. berghei genes with *glmS* should be tolerated.

**FIG 1:**
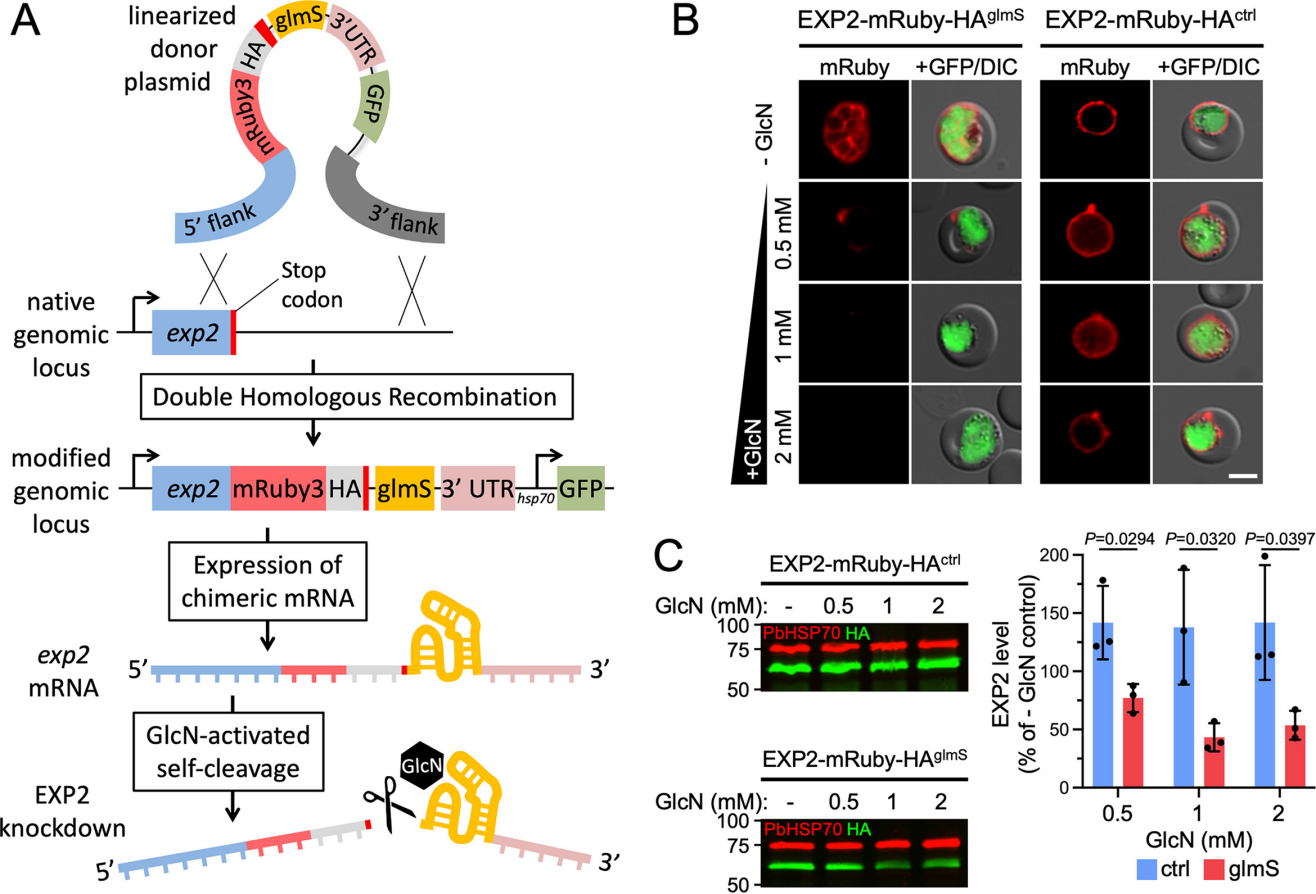
The *glmS* ribozyme mediates EXP2 knockdown in the P. berghei blood stage. (A) Schematic showing the strategy for double homologous recombination to install an mRuby3-3×HA tag followed by the *glmS* sequence immediately downstream of the stop codon at the endogenous *exp2* locus (EXP2-mRuby-HA^glmS^). The construct also contains a downstream cassette for expression of cytosolic GFP. Following transcription, the ribozyme is activated by binding to GlcN-6-phosphate, resulting in autocleavage to remove the mRNA 3′ UTR. A control line, identical except for lacking the *glmS* sequence, was also generated (EXP2-mRuby-HA^ctrl^; not shown). (B) Live fluorescence imaging and (C) Western blot of EXP2-mRuby-HA^glmS^ and EXP2-mRuby-HA^ctrl^ parasites following 18 h of *ex vivo* culture in the absence or presence of the indicated GlcN concentrations. PbHSP70 serves as a loading control. Molecular weights are predicted to be 75 kDa for PbHSP70 and 58.9 kDa for EXP2-mRuby-HA after signal peptide cleavage. Bars = 5 μm. Results are representative of three independent experiments. Quantification of EXP2-mRuby-HA levels is shown. Error bars indicate SD. *P* values were determined by an unpaired, two-sided Student’s *t* test.

To evaluate whether *glmS* can control P. berghei EXP2 levels, we first introduced an mRuby3-3×HA tag followed by the *glmS* sequence at the endogenous P. berghei *exp2* locus by double homologous recombination (Fig. 1A; also, see Fig. S1 in the supplemental material). The donor plasmid also contains a cassette for expression of GFP driven by the constitutive *Pbhsp70* promoter. In parallel, we also generated a control line that was identical except for lacking the *glmS* sequence. Addition of GlcN to *ex vivo* cultures for 18 h substantially reduced EXP2 levels relative to untreated controls in the *glmS* line but did not reduce EXP2 levels in the control parasites lacking the ribozyme (Fig. 1B and C). Specifically, treatment with 0.5 mM GlcN reduced EXP2 levels by 23% ± 12%, while higher concentrations achieved ~50% knockdown (57% ± 12% or 46% ± 12% at 1 or 2 mM GlcN, respectively) (Fig. 1C), similar to previous reports (27). These results indicate the *glmS* ribozyme can modulate P. berghei EXP2 levels, although blood-stage knockdown is less substantial than that by *glmS* in P. falciparum (14), likely due to the inability to initiate GlcN treatment in the preceding cycle of P. berghei development. Furthermore, parasites used to initiate *ex vivo* cultures were not synchronized, and thus, any cells beyond ring-stage development will have already expressed substantial EXP2 prior to ribozyme activation (10).

10.1128/mbio.03096-22.1 FIG S1 Generation of EXP2-mRuby-3×HA *glmS* parasites. (A) Detailed schematic showing modification of the endogenous *exp2* locus by double homologous recombination resulting in a C-terminal mRuby3-3×HA fusion followed by the *glmS* ribozyme immediately downstream of the stop codon in the 3′ UTR. The plasmid also contains a downstream cassette for expression of GFP under the control of the *hsp70* promoter. In parallel, a control parasite line was generated with a plasmid lacking the *glmS* sequence but otherwise identical. (B) Diagnostic PCR with primers indicated in the schematic, showing successful integration at the *exp2* locus. Download FIG S1, JPG file, 0.9 MB. Copyright © 2022 Hussain et al. 2022 Hussain et al. https://creativecommons.org/licenses/by/4.0/ This content is distributed under the terms of the Creative Commons Attribution 4.0 International license.

To determine the suitability of the *glmS* system for protein knockdown in cultured liver-stage parasites, we first evaluated whether GlcN levels sufficient to mediate knockdown in blood-stage parasites had any effect on proliferation of Huh7 or HepG2 hepatoma cell lines commonly used to cultivate the P. berghei liver stage (29). We assessed viability of hepatoma cells by quantifying metabolic activity using a resazurin assay (30) and found that proliferation of both Huh7 and HepG2 cell types was not reduced at GlcN concentrations up to 1 mM (Fig. S2). These results indicate that GlcN can be supplied in hepatoma cultures at concentrations suitable for maximal blood-stage knockdown without host cell toxicity. As HepG2 and Huh7 cells were similarly tolerant to GlcN levels, Huh7 cells were chosen for subsequent experiments due to their superior qualities for imaging intracellular parasites.

10.1128/mbio.03096-22.2 FIG S2 Impact of GlcN on hepatoma cell viability. Dose-response curves of Huh7 and HepG2 hepatoma cells measured using a resazurin assay. Curves were fitted to the mean from four independent biological replicates, each performed in technical triplicate. Error bars indicate standard errors of the means (SEM). The EC_50_ was determined to be 3.7 mM for Huh7 cells and 8 mM for HepG2 cells. Download FIG S2, JPG file, 0.3 MB. Copyright © 2022 Hussain et al. 2022 Hussain et al. https://creativecommons.org/licenses/by/4.0/ This content is distributed under the terms of the Creative Commons Attribution 4.0 International license.

To evaluate whether the *glmS* ribozyme can mediate control of parasite protein levels in the liver stage, we next generated a line bearing a 3×FLAG epitope tag followed by the *glmS* sequence (or not, as a control) at the endogenous P. berghei *exp2* locus. This plasmid also contains a downstream cassette expressing nanoluciferase (NanoLuc) under the control of the *Pbhsp70* promoter to provide a sensitive proxy for monitoring parasite development (31). Clonal lines of each strain were derived and designated EXP2^glmS^ and EXP2^ctrl^, respectively (Fig. S3). These lines showed similar levels of exflagellation, and following blood feeding to mosquitoes, both lines produced numbers of sporozoites per mosquito similar to those seen with parental parasites, indicating that modification of the *exp2* genetic locus did not impact transmission to or development in the insect vector (Fig. S4).

10.1128/mbio.03096-22.3 FIG S3 Generation of EXP2*^glmS^* and EXP2^ctrl^ parasites. (A) Schematic showing modification of the endogenous *exp2* locus by double homologous recombination, resulting in a C-terminal 3×FLAG fusion followed by the *glmS* ribozyme immediately downstream of the stop codon in the 3′ UTR. The plasmid also contains a downstream cassette for expression of nanoluciferase under the control of the *hsp70* promoter. In parallel, a control parasite cell line was generated with a plasmid lacking the *glmS* sequence but otherwise identical. (B) Diagnostic PCR with primers indicated in the schematic, showing successful integration at the *exp2* locus. Download FIG S3, JPG file, 0.8 MB. Copyright © 2022 Hussain et al. 2022 Hussain et al. https://creativecommons.org/licenses/by/4.0/ This content is distributed under the terms of the Creative Commons Attribution 4.0 International license.

10.1128/mbio.03096-22.4 FIG S4 Sporozoite production is unaltered in EXP2*^glmS^* and EXP2^ctrl^ parasites. (A) Quantification of exflagellation centers 5 days after infection with EXP2*^glmS^* and EXP2^ctrl^ parasites. (B) Quantification of sporozoites obtained per mosquito following dissection 21 days after blood feeding for the indicated parasite lines. Data points represent independent biological replicates. Error bars indicate SD. Download FIG S4, JPG file, 0.4 MB. Copyright © 2022 Hussain et al. 2022 Hussain et al. https://creativecommons.org/licenses/by/4.0/ This content is distributed under the terms of the Creative Commons Attribution 4.0 International license.

**FIG 2:**
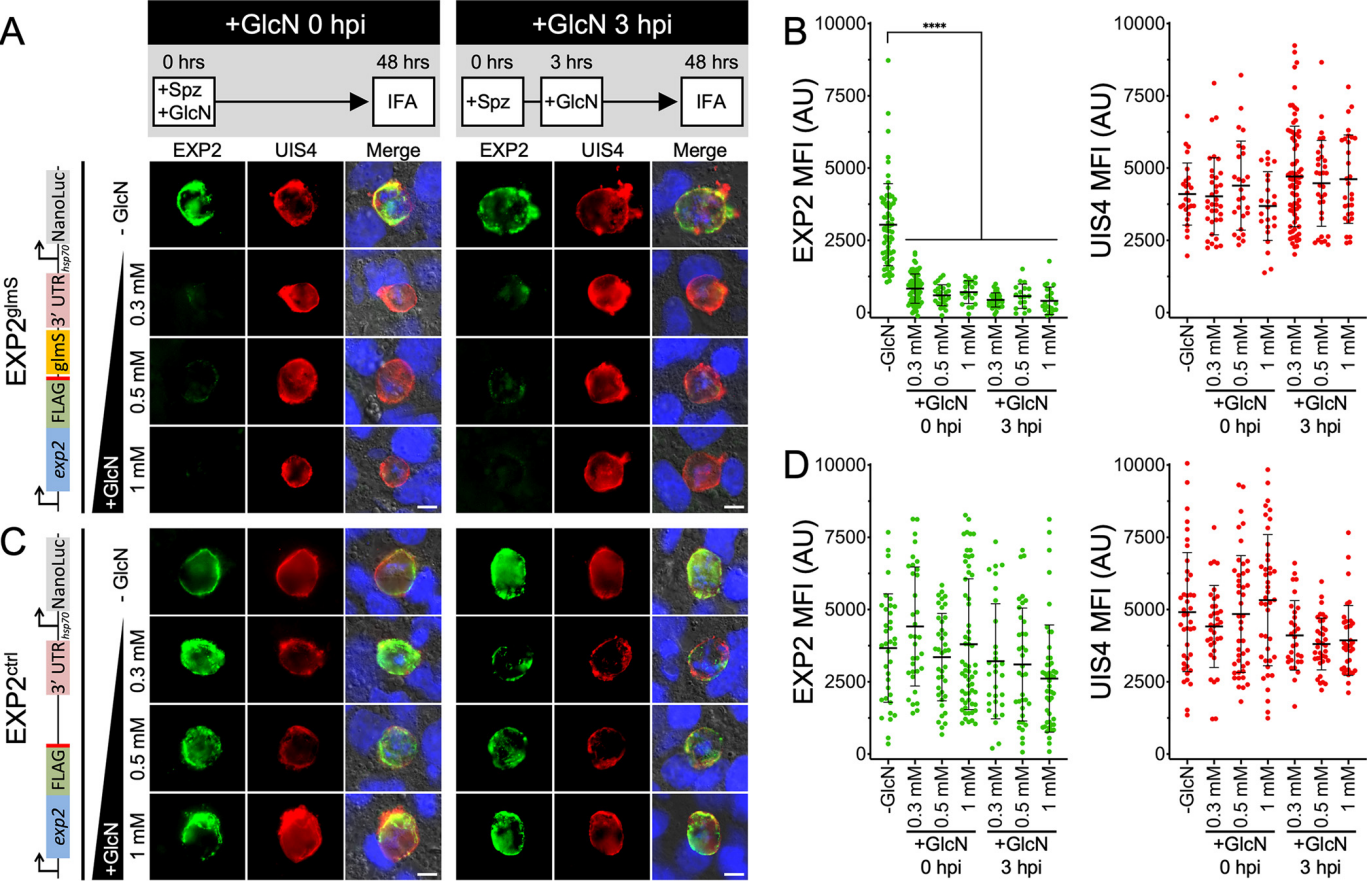
The *glmS* ribozyme mediates EXP2 knockdown in the P. berghei liver stage. (A) Immunofluorescence assay on EXP2^glmS^ liver-stage parasites after 48 h of development in Huh7 cells detecting EXP2 (FLAG) in green and UIS4 in red. GlcN was added at the indicated concentrations at the time sporozoites were introduced (0 hpi) or 3 h later after sporozoites had invaded (3 hpi) as outlined in the schematic at the top. Merged panels also show DAPI (blue) and differential inference contrast (DIC). The schematic at left shows the modified *exp2* locus. Images are representative of three independent experiments. Bars = 5 μm. Spz, sporozoites. (B) Quantification of EXP2 and UIS4 intensity. UIS4 was used to mark the PVM, and EXP2-3×FLAG and UIS4 mean fluorescence intensity (MFI) within this area was collected. Data are pooled from three independent experiments. Error bars represent standard deviations (SD). AU, arbitrary units. (C) Immunofluorescence assay on EXP2^ctrl^ liver-stage parasites and (D) quantification of EXP2 and UIS4 intensity in EXP2^ctrl^ parasites as described for panel B. Significance was determined by an unpaired, two-sided Student’s *t* test. ****, *P* < 0.0001.

EXP2^glmS^ and EXP2^ctrl^ sporozoites were isolated by mosquito dissection and allowed to infect Huh7 cells and develop in the presence or absence of 0.3 mM, 0.5 mM, or 1 mM GlcN for 48 h before cells were fixed and processed for immunofluorescence. In untreated EXP2^glmS^ cultures, EXP2 was readily observed at the parasite periphery, where it largely colocalized with the liver-stage integral PVM marker UIS4 (32) (Fig. 2A, −GlcN [top panels]). In contrast, EXP2^glmS^ parasites grown with GlcN displayed a striking reduction in EXP2 intensity, ~73 to 87%, while UIS4 levels were not affected (Fig. 2A and B). Importantly, EXP2 levels in the control line were not similarly altered by GlcN, indicating that the reduction observed in the EXP2^glmS^ parasites was a result of the ribozyme sequence in the *exp2* 3′ UTR and not an indirect effect of GlcN treatment (Fig. 2C and D). Notably, introduction of 0.5 mM GlcN at the time when EXP2^glmS^ sporozoites were added to Huh7 host cells (0 h postinfection [hpi]) had no impact on invasion (Fig. S5) but reduced EXP2 levels by ~80% at 48 hpi when maintained in cultures (Fig. 2B). Moreover, similar EXP2 knockdown was achieved when GlcN was added after sporozoite invasion had occurred (3 hpi) (Fig. 2A and B), clearly indicating successful EXP2 depletion after host cell entry without confounding effects from perturbed invasion. Collectively, these results show that the *glmS* ribozyme can be activated in the liver stage, enabling temporal control of parasite protein knockdown.

10.1128/mbio.03096-22.5 FIG S5 GlcN treatment does not impact EXP2^glmS^ sporozoite invasion. Sporozoites from EXP2^glmS^ parasites were allowed to infect Huh7 cells in the presence or absence of 0.5 mM GlcN for 3 h before monolayers were washed, fixed, and processed for IFA. Images were acquired on an automated Keyence microscope, and UIS4-positive vacuoles per square millimeter were quantified. Data from 3 independent biological replicates are shown. Error bars indicate SD. Download FIG S5, JPG file, 0.1 MB. Copyright © 2022 Hussain et al. 2022 Hussain et al. https://creativecommons.org/licenses/by/4.0/ This content is distributed under the terms of the Creative Commons Attribution 4.0 International license.

### EXP2 knockdown reduces liver-stage parasite size and severely impacts intrahepatic parasite development.

**FIG 3:**
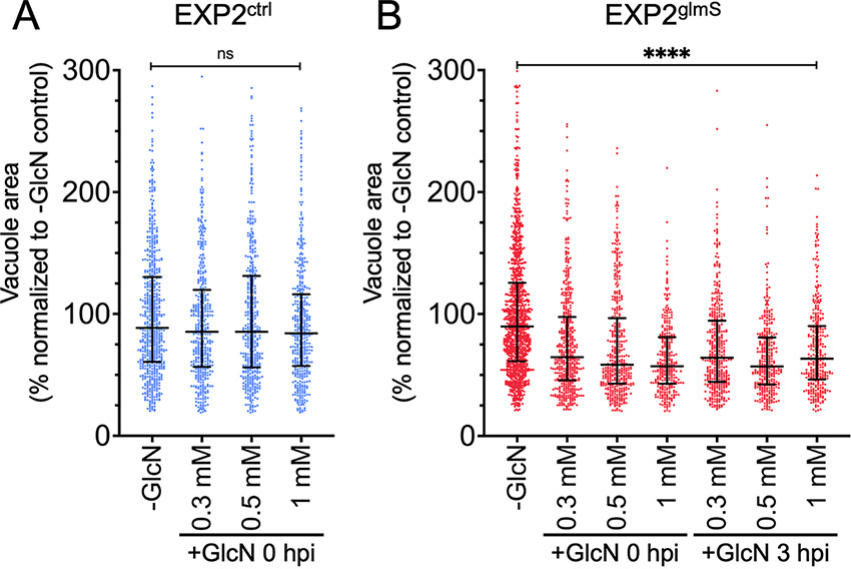
EXP2 knockdown reduces liver-stage parasite size. Quantification of parasite vacuolar area at 48 hpi in (A) EXP2^ctrl^ or (B) EXP2^glmS^. GlcN was introduced to cultures at the indicated concentrations and times. Data are pooled from three independent experiments and expressed as percentages of the mean vacuole area in the untreated controls. Medians and interquartile ranges are shown. *P* values were determined by a Kruskal-Wallis test. ****, *P* < 0.0001; ns, not significant.

To determine the impact of EXP2 knockdown on intrahepatic parasite development, EXP2^ctrl^ and EXP2^glmS^ parasites were allowed to develop in Huh7 cells for 48 h in the presence or absence of 0.3, 0.5, or 1 mM GlcN, and vacuole size was determined by automated microscopy using UIS4 as a marker for the PVM (Fig. S6). While EXP2^ctrl^ parasite size was not altered by GlcN treatment (Fig. 3A), EXP2^glmS^ parasites showed a significant decrease in vacuole area at all GlcN treatment levels, with mean vacuole size reduced by as much as 36% (Fig. 3B, +1 mM GlcN at 0 hpi). A similar decrease in parasite size was observed when GlcN was not added until after sporozoite invasion had occurred (Fig. 3B, +GlcN at 3 hpi).

10.1128/mbio.03096-22.6 FIG S6 Representative images used for quantification of vacuolar area. Sporozoites were obtained by mosquito dissection and allowed to infect Huh7 monolayers for 3 h before a medium change to remove remaining extracellular parasites. GlcN was added at the indicated concentrations from the time sporozoites were introduced to Huh7 cultures (0 hpi) or following the initial medium change (3 hpi). Medium was changed daily, and cells were fixed and processed for IFA at 48 hpi. Images were acquired on an automated Keyence microscope using a 40× objective and analyzed to determine vacuolar area using UIS4 to mark the PVM. Representative fields are shown. Bar = 62.5 μm. Download FIG S6, JPG file, 1.4 MB. Copyright © 2022 Hussain et al. 2022 Hussain et al. https://creativecommons.org/licenses/by/4.0/ This content is distributed under the terms of the Creative Commons Attribution 4.0 International license.

**FIG 4:**
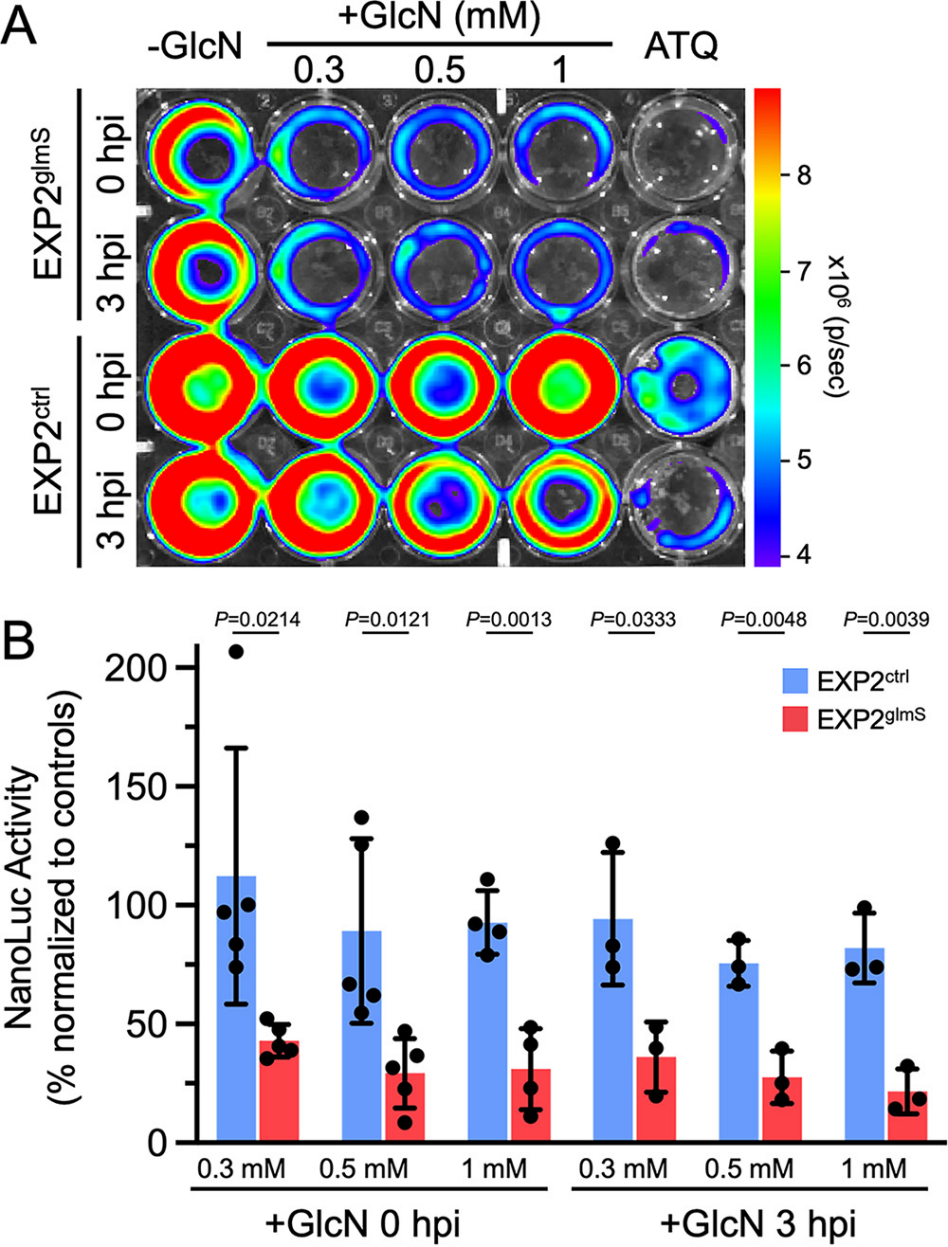
EXP2 knockdown severely impacts a liver-stage bioluminescence developmental reporter. (A) Representative IVIS image of NanoLuc luminescence in Huh7 cultures 48 hpi with EXP2^glmS^ and EXP2^ctrl^. GlcN was introduced to cultures at the indicated concentrations and times. Atovaquone (ATQ; 10 nM) was added at 0 hpi as a control for parasite death. (B) Quantification of luminescence in EXP2^glmS^ and EXP2^ctrl^ (red and blue bars, respectively). Data were normalized to untreated (−GlcN) and ATQ controls (used to define 100% and 0% NanoLuc activity, respectively, in each experiment). *n* = 3 to 5 independent biological replicates. Error bars indicate SD. *P* values were determined by an unpaired, two-sided Student’s *t* test.

To provide an orthogonal, ultrasensitive measurement of parasite development, we next monitored luminescence generated from the constitutive NanoLuc expression cassette installed downstream of *exp2* (Fig. S3A). Parasites were again allowed to develop for 48 h in the presence or absence of 0.3, 0.5, or 1 mM GlcN before luminescence of the infected monolayer was measured. As a control for complete parasite death, we also included an atovaquone treatment (33). Luminescence in EXP2^glmS^ cultures exposed to GlcN was strikingly reduced by as much as 78.4% ± 9.4% (with 1 mM GlcN at 3 hpi), but the NanoLuc signal in EXP2^ctrl^ parasites was not substantially impacted (Fig. 4). Importantly, a similar reduction in EXP2^glmS^ luminescence was observed regardless of whether GlcN was added at 0 or 3 hpi, indicating that this effect was not the result of a reduction in sporozoite invasion.

**FIG 5:**
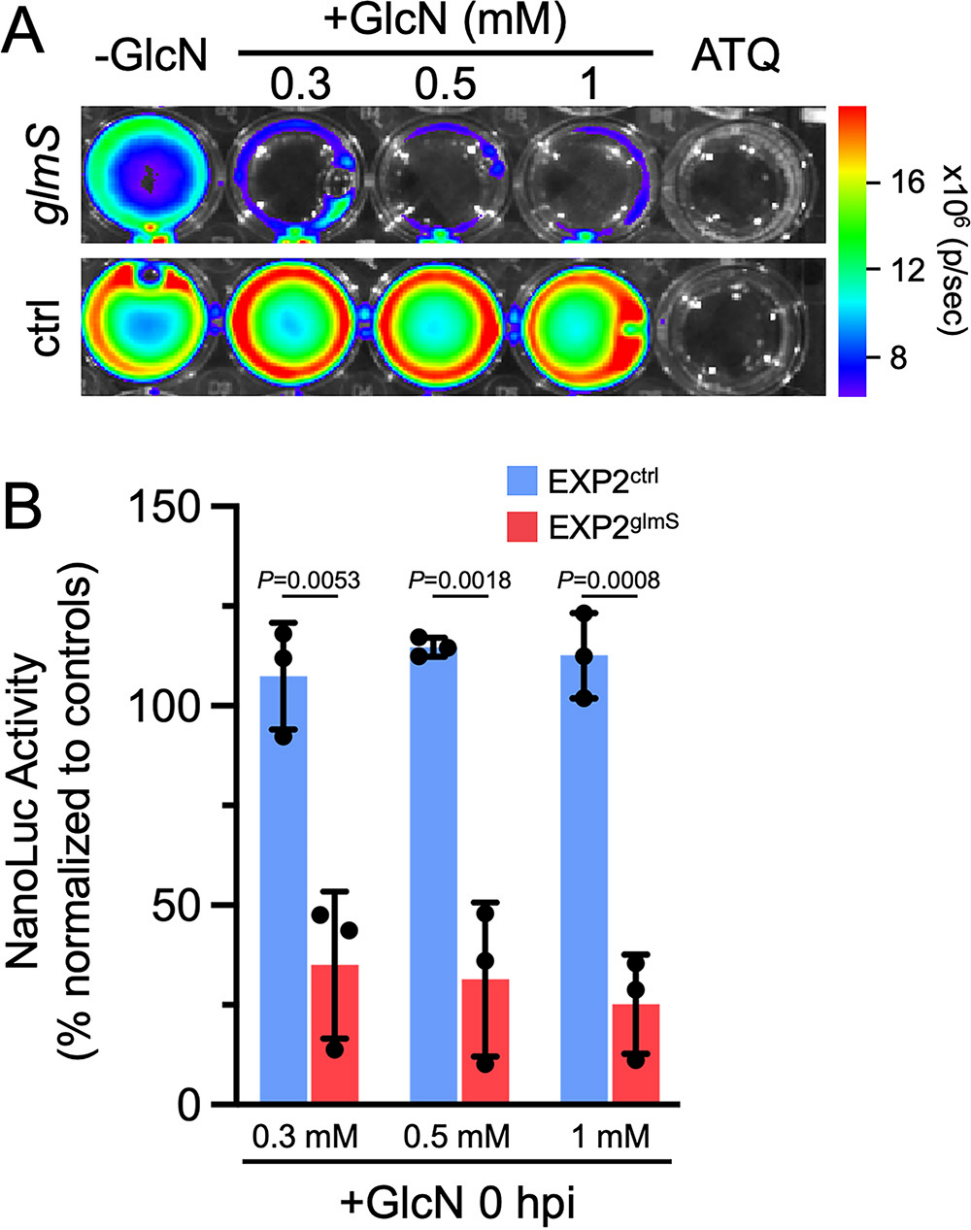
EXP2 knockdown reduces the formation of detached merosomes. (A) Representative IVIS image of NanoLuc luminescence in Huh7 culture supernatants at 65 hpi with EXP2^glmS^ and EXP2^ctrl^. GlcN was introduced into cultures at the indicated concentrations at 0 hpi. Atovaquone (ATQ; 10 nM) was added at 0 hpi as a control for parasite death. (B) Quantification of luminescence in EXP2^glmS^ and EXP2^ctrl^ (red and blue bars, respectively). Data were normalized to untreated (−GlcN) and ATQ controls (used to define 100% and 0% NanoLuc activity, respectively, in each experiment). *n* = 3 independent biological replicates. Error bars indicate SD. *P* values were determined by an unpaired, two-sided Student’s *t* test.

Liver-stage development culminates in detachment of infected cells from the liver matrix as merosomes, which enter the circulation to initiate the blood stage, a process that is recapitulated in culture by detachment of merosomes from the monolayer and release into the medium (31, 34, 35). To determine if parasites depleted of EXP2 were able to complete the liver stage and form merosomes, we measured NanoLuc signal in culture supernatants at 65 hpi. While luminescence was not impacted by GlcN treatment in EXP2^ctrl^ cultures, indicating productive merosome release, EXP2^glmS^ parasites showed a severe reduction in supernatant luminescence by as much as 74.9% ± 12.5% (with 1 mM GlcN), indicating that loss of EXP2 has a major impact on successful completion of the liver stage (Fig. 5). Collectively, these results show that EXP2 is important for intrahepatic parasite development independent of any contribution to host cell entry.

### EXP2 knockdown decreases expression of the liver-stage developmental marker LISP2.

**FIG 6:**
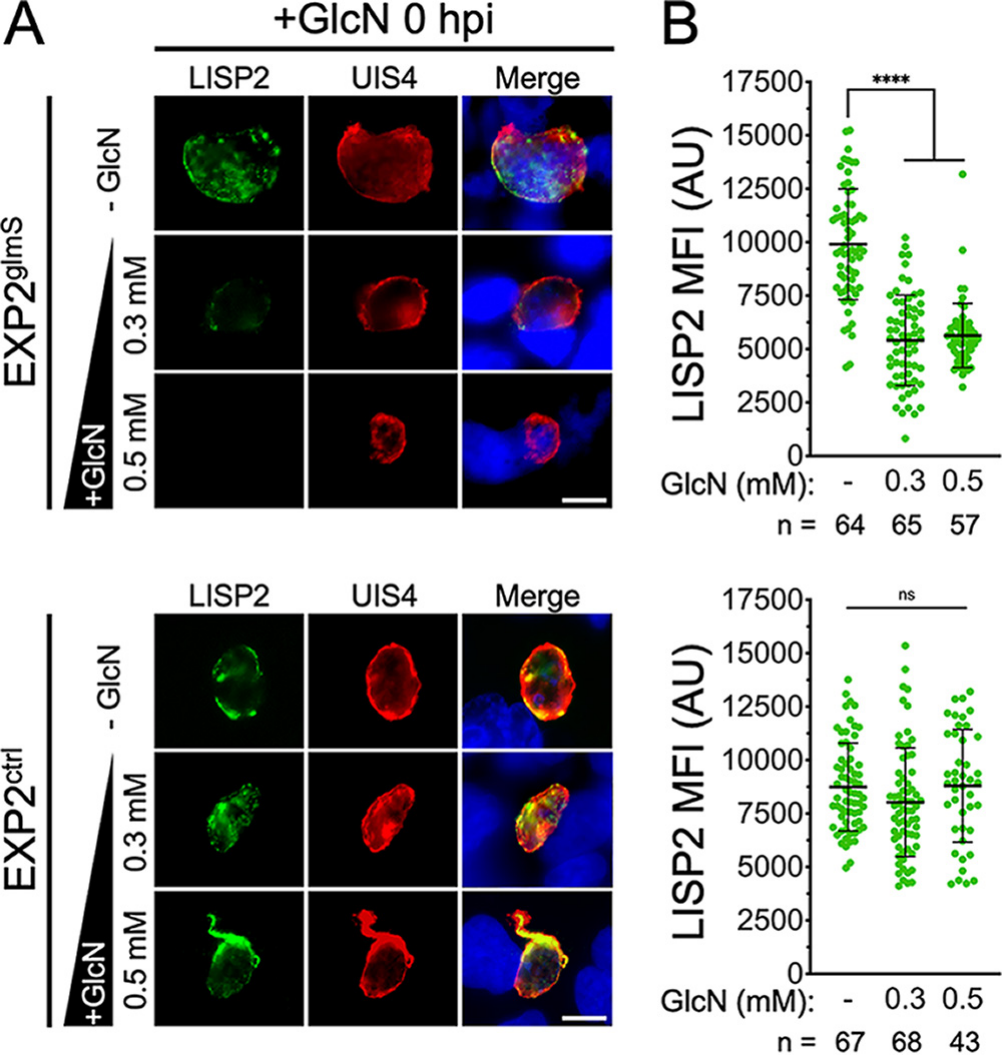
EXP2 knockdown abrogates expression of the mid- to late-liver-stage developmental marker LISP2. (A) Immunofluorescence assay on EXP2^glmS^ and EXP2^ctrl^ parasites after 48 h of development in Huh7 cells detecting LISP2 and UIS4. GlcN was added at the indicated concentrations at the time sporozoites were introduced (0 hpi). Merged panels also show DAPI (blue). Bars = 5 μm. (B) Quantification of LISP2. UIS4 was used to mark the PVM, and LISP2 mean fluorescence intensity (MFI) within this area was collected. Data are pooled from three independent experiments. Error bars represent SD. Significance was determined by an unpaired, two-sided Student’s *t* test. ****, *P* < 0.0001; ns, not significant.

To better understand when liver-stage defects occur following EXP2 knockdown, we examined expression of liver-specific protein 2 (LISP2). LISP2 is expressed from the midpoint of development (beginning ~24 hpi in P. berghei) through the end of schizogony, providing a marker for transition into the later stages of liver-stage development (36–39). We quantified LISP2 expression levels at 48 hpi in EXP2^glmS^ and EXP2^ctrl^ parasites with or without 0.3 or 0.5 mM GlcN treatment using LISP2 antisera raised against a peptide near the beginning of the C-terminal 6-Cys domain that localizes to the PV (36, 39). While LISP2 levels were not significantly altered by GlcN treatment in EXP2^ctrl^ parasites, EXP2^glmS^ parasites displayed a striking loss of LISP2 expression in the presence of GlcN, with the majority of cells showing no apparent LISP2 signal (Fig. 6). Loss of LISP2 expression suggests that EXP2 is important for early liver-stage development and that parasites depleted of EXP2 do not proceed normally into schizogony, consistent with an anticipated function in nutrient uptake and/or protein export across the PVM.

## DISCUSSION

Nutrient transport and effector protein translocation across the PVM are critical for the malaria parasite to establish its intracellular niche in the erythrocyte, avoid host defenses, and fuel rapid parasite growth (40). Though less well understood, similar transport mechanisms are expected to be in operation at the hepatocyte-parasite interface during liver-stage infection. Indeed, the liver-stage vacuole is also permeable to small molecules (41), consistent with the presence of a nutrient-permeable channel similar to that observed in the blood stage and in related apicomplexans (42–44). To promote their growth, liver-stage *Plasmodium* parasites also recruit hepatocyte factors to the host-parasite interface, including the water/glycerol channel aquaporin 3 (45–47) and the endosomal adaptor APPL1 (48). More broadly, parasites manipulate host gene expression (49, 50), inhibit apoptotic cell death (35), and remodel host cell biology (51) while avoiding hepatocyte cellular defenses such as vacuole-targeted selective autophagy (52, 53). Evasion of autophagy-related defenses depends on the PVM protein UIS3, which sequesters host LC3 (54–56), and other effectors secreted beyond the PV are likely to be involved in hepatocyte subversion.

While nutrient transport and protein export across the intraerythrocytic PVM both depend on the PTEX membrane pore EXP2 (10, 14), the intrahepatic function of EXP2/PTEX has remained unclear. A recent study unexpectedly reported that EXP2 is secreted by extracellular sporozoites and is important for hepatocyte invasion to initiate the liver stage (22). As EXP2 knockdown in the blood stage was not observed to impact erythrocyte invasion (10, 14), this additional EXP2 function is apparently unique to hepatocyte entry. However, whether EXP2 performs important roles in the PV during intrahepatic development has remained obscure. Here, we show that the *glmS* ribozyme can be used to control parasite protein expression in the liver stage, enabling exploration of EXP2 function during intracellular development in hepatoma cells.

The ribozyme self-cleavage activity of *glmS* is predominantly activated by GlcN-6-phosphate (GlcN-6-P) binding and, to a lesser extent, by unphosphorylated GlcN and related amine-containing molecules (57). GlcN-6-P is generated from fructose-6-phosphase at the glycolytic branch point that feeds into the hexosamine biosynthesis pathway or by uptake and phosphorylation of GlcN from the environment. Differences in hexosamine metabolic flux between hepatoma and red blood cells notwithstanding, we found that basal Huh7 GlcN-6-P levels were not sufficient to produce EXP2 knockdown without addition of exogenous GlcN (Fig. 2B and D). In contrast, GlcN supplementation produced a robust and specific knockdown in EXP2^glmS^ parasites, reducing EXP2 levels by ~75% and demonstrating ribozyme-mediated control of gene expression.

While similar GlcN concentrations activated EXP2 knockdown in blood- and liver-stage parasites, knockdown experiments in the blood stage showed a more substantial dose response to GlcN concentrations than the liver-stage knockdown. GlcN transport into mammalian cells occurs primarily through GLUT2, the major hexose transporter expressed in hepatocytes, which has uniquely high affinity for GlcN over other substrates, but also via GLUT4 or GLUT1, the predominant hexose transporter expressed in erythrocytes (58–61). Additionally, hepatoma cells show alterations in transporter expression and metabolism compared to primary cells (62, 63), and GLUT1 activity is also increased in cells infected with liver-stage P. berghei (64). Thus, knockdown sensitivity could reflect differences in host cell GlcN uptake or transport across the parasite plasma membrane; alternatively, this might result from the longer incubation times afforded by the intrahepatic parasite developmental time frame as well as the more synchronous nature of the liver-stage experiments that allows uniform induction of knockdown immediately after invasion. To our knowledge, this is the first example of *glmS* utilization to control protein expression in an intracellular pathogen within a nucleated host cell, extending its range of application and providing a new tool to allow temporal control of protein knockdown in the liver stage.

Invasion of EXP2^glmS^ sporozoites was not impacted when GlcN and parasites were introduced simultaneously into Huh7 cultures (Fig. S5), possibly due to insufficient time to achieve substantial mRNA degradation. In contrast, intrahepatic EXP2 levels could be depleted even when GlcN was not added until after sporozoite invasion, allowing EXP2 functional analysis in intracellular liver-stage parasites without confounding effects on invasion. Analysis of vacuole size and NanoLuc reporter luminescence (as a proxy for parasite growth and maturation) clearly indicate that EXP2 is important for intrahepatic development (Fig. 3 to 5). Furthermore, loss of LISP2 expression following EXP2 knockdown indicates a defect at an early stage, consistent with the established blood-stage roles of EXP2 in protein export and nutrient uptake that are critical to early parasite development in the erythrocyte (10, 14). In *Toxoplasma*, the EXP2 orthologs GRA17 and -23 function in PVM permeation but not in an analogous but mechanistically distinct PV protein export pathway; thus, it is possible that EXP2 might function exclusively in small-molecule transport during the liver stage (13, 40). The apparent absence of HSP101 in the liver-stage PV and the observation that several parasite proteins or reporters which are exported into the erythrocyte are not similarly translocated into the hepatocyte indicate that protein export is at least mechanistically distinct in the liver stage (15, 16, 18–21). However, the upregulation of PTEX150 in activated sporozoites (65) and its presence together with EXP2 in the liver-stage PV (17) suggest that EXP2 is also functionalized for the protein translocon in the hepatocyte, although it is unclear at present how this minimal translocon would be powered.

While hundreds of putative exported proteins have been identified in the blood stage, very little is known about the effector arsenal secreted into the hepatocyte, with LISP2 being one of only a few parasite proteins reported to cross the PVM (36, 37). LISP2 is a large, conserved *Plasmodium* protein that contains a signal peptide and bona fide PEXEL motif followed by a repeat region and C-terminal 6-Cys domain (36, 66, 67). While the C-terminal portion of LISP2 detected by the antibody used in this study has not been observed to traffic beyond the PV, an N-terminal portion of the protein has been reported to be exported into the hepatocyte cytosol and nucleus, particularly in later stages of development (36 hpi or beyond in P. berghei) (36, 37). Unfortunately, the loss of LISP2 expression in parasites depleted of EXP2 prevented us from evaluating whether EXP2 was important for LISP2 translocation into the host cell. The identification of novel liver-stage exported proteins expressed early during infection along with determination of the importance of PTEX150 to liver-stage development will help clarify the mechanism of protein export into the hepatocyte. Collectively, our findings extend the utility of the *glmS* ribozyme to study protein function in the liver stage and reveal an important function for EXP2 in intrahepatic parasite development, opening the door to studying liver-stage PVM transport mechanisms and their role in hepatocyte subversion.

## MATERIALS AND METHODS

### Parasite maintenance and sporozoite production.

P. berghei ANKA clone 2.34 and derivatives were maintained in Swiss Webster mice (Charles River). To enhance the production of parasite numbers, mosquito infections were performed in a TEP1 mutant strain (Δct1) of Anopheles gambiae (68, 69). Mosquitoes were reared at 27°C and 80% humidity on a 14-h/10-h day/night cycle. Larvae were fed TetraMin (Tetra), and adults were maintained on 10% sucrose. For parasite infection, mosquitoes (4 to 6 days postemergence) were fed on infected mice when parasitemia reached ~5% and exflagellation was observed. Blood-fed mosquitoes were maintained on 10% sucrose at 21°C for 21 to 28 days before isolation of salivary gland sporozoites. All experiments involving rodents were reviewed and approved by the Iowa State University Institutional Animal Care and Use Committee.

### Genetic modification of P. berghei.

Primer sequences are given in Table S1. For generation of EXP2-mRuby3-3×HA *glmS* and control lines, 5′ and 3′ flanking sequences targeting the 3′ end of *exp2* were amplified from parasite genomic DNA using primer pairs P1/2 and P3/4 and inserted between AvrII/XhoI and SacII/HpaI sites, respectively, in the plasmid pBAT1 (70). The mRuby3-3×HA coding sequence was then amplified with P5/6 and inserted between HpaI and SphI sites. A 315-bp sequence containing the *glmS* ribozyme was amplified from plasmid pL6-3HA-glmS-BSD (71) with primers P7 and P8 and inserted at the SphI site between the stop codon and the downstream PbPPPK-DHPS 3′ UTR, resulting in the plasmid pBAT-EXP2-mRuby3-3×HA-*glmS*. To generate a matched control lacking only the ribozyme, the 186 bp comprising the *glmS* sequence were then removed using a QuikChange Lightning Multi site-directed mutagenesis kit (Agilent) and primer P9, resulting in the plasmid pBAT-EXP2-mRuby3-3×HA-control.

For generation of EXP2-3×FLAG *glmS* and control lines, sequence encoding a 3×FLAG tag was inserted between HpaI and SphI sites in pBAT-EXP2-mRuby3-3×HA-*glmS* using P10, replacing mRuby3-3×HA. The NanoLuc coding sequence was then inserted between the SwaI and BamHI sites using P11/12, replacing GFP and placing it under the control of the constitutive *Pbhsp70* promoter, resulting in the plasmid pBAT-EXP2-3×FLAG-*glmS*. To generate a matched control, the *glmS* sequence was then removed from this plasmid using a QuikChange Lightning Multi site-directed mutagenesis kit and primer P9 as described above, resulting in the plasmid pBAT-EXP2-3×FLAG-control.

Plasmids were linearized at the SacII/XhoI sites and transfected into P. berghei as described elsewhere (72), except that schizont-infected RBCs were purified on an LD column mounted on a QuadroMACs magnetic separator (Miltenyi Biotech). Transfected parasites were reinjected into naive mice, and selection was applied with 0.07 mg/mL pyrimethamine in drinking water provided *ad libitum* 24 h later. After returning from selection, transfected populations were cloned by limiting dilution intravenous injection into naive mice to generate isogenic populations.

### GlcN treatment and evaluation of EXP2 knockdown.

For blood-stage knockdown experiments, parasite-infected blood was collected by cardiac puncture and cultured in complete RPMI with or without GlcN supplementation. After 18 h of culture, parasites were imaged by live fluorescence microscopy and collected for Western blotting. For analysis of EXP2 knockdown by Western blotting, parasites were harvested in cold phosphate-buffered saline (PBS) containing 0.035% saponin (Sigma), washed in PBS, and lysed in radioimmunoprecipitation assay (RIPA) buffer containing Halt protease inhibitor cocktail (Thermo Fisher). Lysates were briefly bath sonicated before centrifugation to pellet hemozoin. Cleared supernatants were lysed in sample buffer containing dithiothreitol and boiled before separation by SDS-PAGE. Primary antibodies were detected with IRDye 680- or 800-conjugated secondary antibodies (LI-COR Biosciences) used at 1:10,000. Western blot imaging was performed with an Odyssey infrared imaging system (LI-COR Biosciences), and signal quantification was carried out with Image Studio software (LI-COR Biosciences). Uncropped Western blots are shown in Fig. S7.

For liver-stage knockdown experiments, Huh7 cells were grown in Dulbecco’s modified Eagle medium (DMEM) supplemented with 10% fetal bovine serum (FBS) and penicillin-streptomycin-glutamine (Gibco). Prior to infection, medium was further supplemented with 2.5 μg/mL amphotericin B. Cells were seeded in 24-well plates or on coverslips and allowed to reach 60 to 80% confluence. To initiate infections, 150,000 freshly dissected sporozoites were centrifuged at 300 × *g* for 5 min onto host cells and allowed to invade for 3 h, after which medium was replaced to remove uninfected sporozoites and subsequently changed 3 times per day. GlcN was introduced at the time of sporozoite addition (0 hpi) or at the 3-h medium change (3 hpi).

For immunofluorescence (IFA) assays, cells were fixed with 4% paraformaldehyde for 15 min, permeabilized with PBS containing 0.1% Triton X-100 for 10 min, and blocked in PBS containing 5% bovine serum albumin (BSA) for 1 h. Blocked samples were incubated with primary antibodies diluted as indicated below for 1 h followed by extensive washing. Primary antibodies were detected by incubation with Alexa Fluor 488- or 594-conjugated secondary IgG antibodies (Thermo Fisher) used at 1:2,000 for 1 h; following extensive washing, coverslips were mounted in ProLong Gold containing DAPI (4′,6-diamidino-2-phenylindole; Thermo Fisher). Live fluorescence and immunofluorescence images for display were collected with a 63× objective on an Axio Observer 7 microscope equipped with an AxioCam 702 mono camera and Zen 2.6 Pro software (Zeiss) using the same exposure times for all images across sample groups and experimental replicates. For quantification of EXP2 expression and vacuolar area, images were collected using a 40× objective on a BZ-X810 automated microscope (Keyence). The thresholding tool was applied to the UIS4 channel to define a region of interest (ROI) corresponding to the boundary of each vacuole while investigators were blind to all other channels. The EXP2 and UIS4 or LISP2 mean fluorescence intensity within each ROI was then collected along with the area of each ROI. Vacuolar areas were normalized and plotted as percentage of the mean area of untreated controls.

### Sporozoite invasion assays.

EXP2^glmS^ sporozoites were allowed to infect Huh7 cells in the presence or absence of 0.5 mM GlcN as described above. After 3 h, medium was changed before coverslips were fixed and processed for IFA. Images were acquired as described above on a Keyence BZ-X810 automated microscope, and parasites that had invaded a host cell were detected by thresholding UIS4 signal and area to distinguish newly formed vacuoles from noninvaded sporozoites to determine the number of successful invasions per sample area.

### Bioluminescence assays.

Quantification of NanoLuc signal was carried out using a Nano-Glo luciferase assay kit (Promega) according to the manufacturer’s recommendations. To monitor development in attached cells, 48-hpi liver-stage cultures were washed with PBS and lysed in Nano-Glo luciferase assay buffer containing Nano-Glo luciferase assay substrate diluted 1:200, and luminescence was measured after 3 min on an IVIS SpectrumCT imaging system (PerkinElmer). To monitor merosome detachment at 65 hpi, parasite culture was carried out as described above until 40 hpi, when medium was changed for a final time and the volume was reduced to 200 μL. At 65 hpi, medium was collected and transferred to new plate, and luminescence was recorded as described above except that the Nano-Glo luciferase assay substrate was diluted 1:100 into the assay buffer. In each case, uninfected Huh7 cells were used as a negative control. To control for variance between independent sporozoite batches across biological replicates, luminescence signals were normalized to the untreated, 0 mM GlcN samples in each experiment. Data were then pooled and plotted as percent NanoLuc activity normalized to the mean luminescence in untreated, 0 mM GlcN controls (100%) and atovaquone-treated controls (0%).

### Antibodies.

The following antibodies were used for IFA and Western blotting at the indicated dilutions: rabbit polyclonal anti-HA SG77 (Thermo Fisher; 1:1,000 [WB]), mouse anti-PbHSP70 monoclonal antibody 4C9 (73) (1:1,000 [WB]), mouse anti-FLAG monoclonal antibody M2 (Sigma; 1:1,000 [IFA]), goat polyclonal anti-UIS4 (LS Biolabs LS-C204260; 1:1,000 [IFA]), and rabbit polyclonal anti-LISP2 (1:1,000 [IFA]). Anti-LISP2 antiserum was generated by GenScript against the peptide NGQKGNVDEERKSM\, located between the repeat region and 6-Cys domain, a portion of LISP2 previously shown to localize to the PV but not observed to enter the host cell (39). Purified antiserum labeled the parasite periphery in immunofluorescence assays, and loss of signal following EXP2 knockdown provided validation of specificity.

### Resazurin assays.

Huh7 or HepG2 cells were seeded at 4,000 cells/well in black-walled, clear-bottom 96-well plates and cultured in complete DMEM supplemented with a series of GlcN concentrations. After 72 h, 44 μM resazurin was added for 5 h before fluorescence was recorded (excitation and emission wavelengths, 530 and 585 nm) on a CLARIOstar Plus plate reader (BMG Labtech). Data were expressed as percent growth following normalization to minimum and maximum values. Dose-response curves were fitted to the mean of four independent biological replicates, and half maximal effective concentrations (EC_50_s) were derived using Prism v9 (GraphPad).

10.1128/mbio.03096-22.7 FIG S7 Uncropped Western blots for quantification of EXP2 knockdown in the *ex vivo* blood-stage experiments whose results are shown in Fig. 1. Download FIG S7, JPG file, 0.5 MB. Copyright © 2022 Hussain et al. 2022 Hussain et al. https://creativecommons.org/licenses/by/4.0/ This content is distributed under the terms of the Creative Commons Attribution 4.0 International license.

10.1128/mbio.03096-22.8 TABLE S1 Sequences of primers used in this study. Download Table S1, XLSX file, 0.01 MB. Copyright © 2022 Hussain et al. 2022 Hussain et al. https://creativecommons.org/licenses/by/4.0/ This content is distributed under the terms of the Creative Commons Attribution 4.0 International license.
