# Supplementary figures and images for: The PTEX Pore Component EXP2 Is Important for Intrahepatic Development during the Plasmodium Liver Stage
Source: mBio. 2022 Nov 29;13(6):e03096-22. doi: 10.1128/mbio.03096-22 (PMC9765067; doi:10.1128/mbio.03096-22)

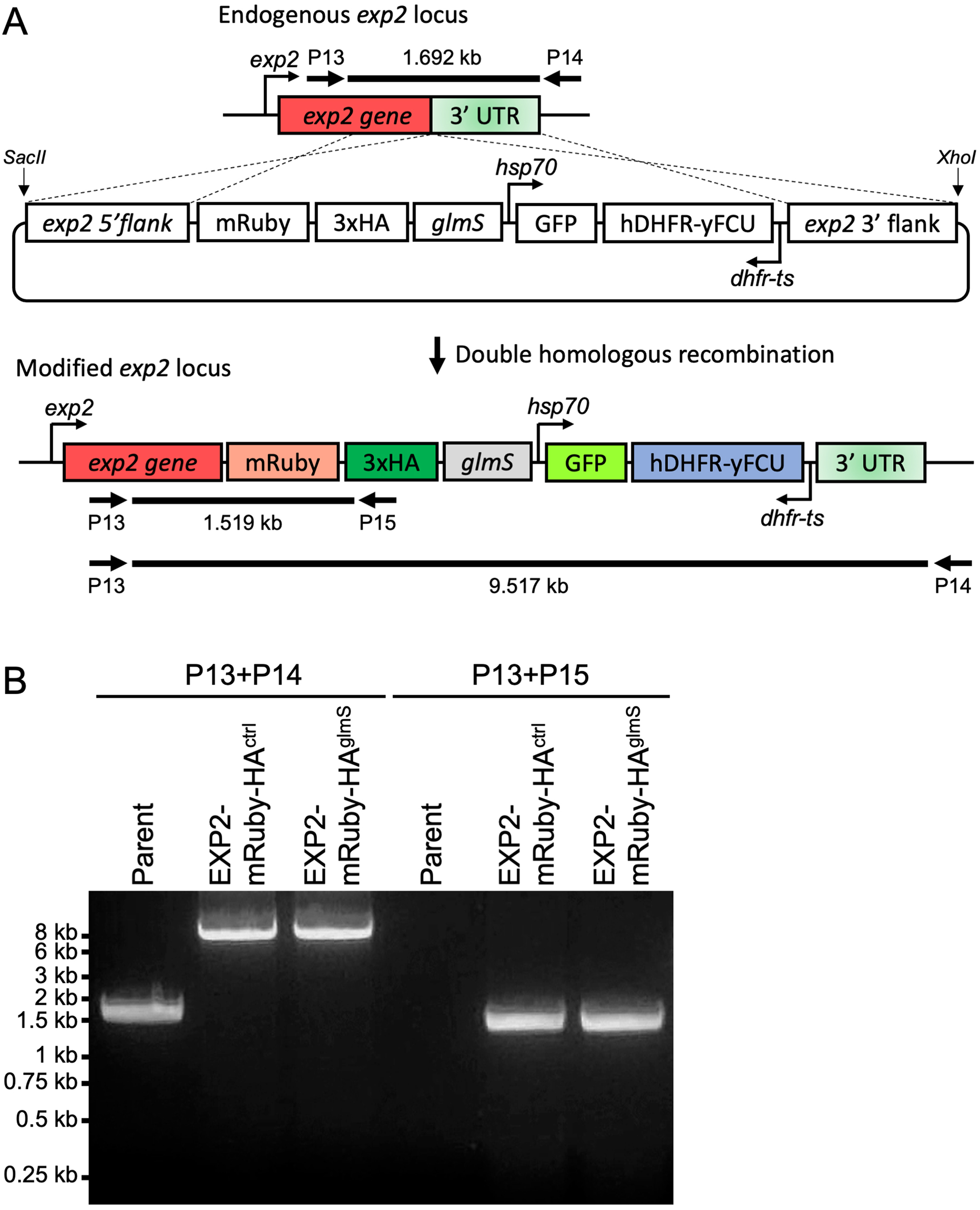

Supplement: FIG S1 [file mbio.03096-22-s0001.jpg]

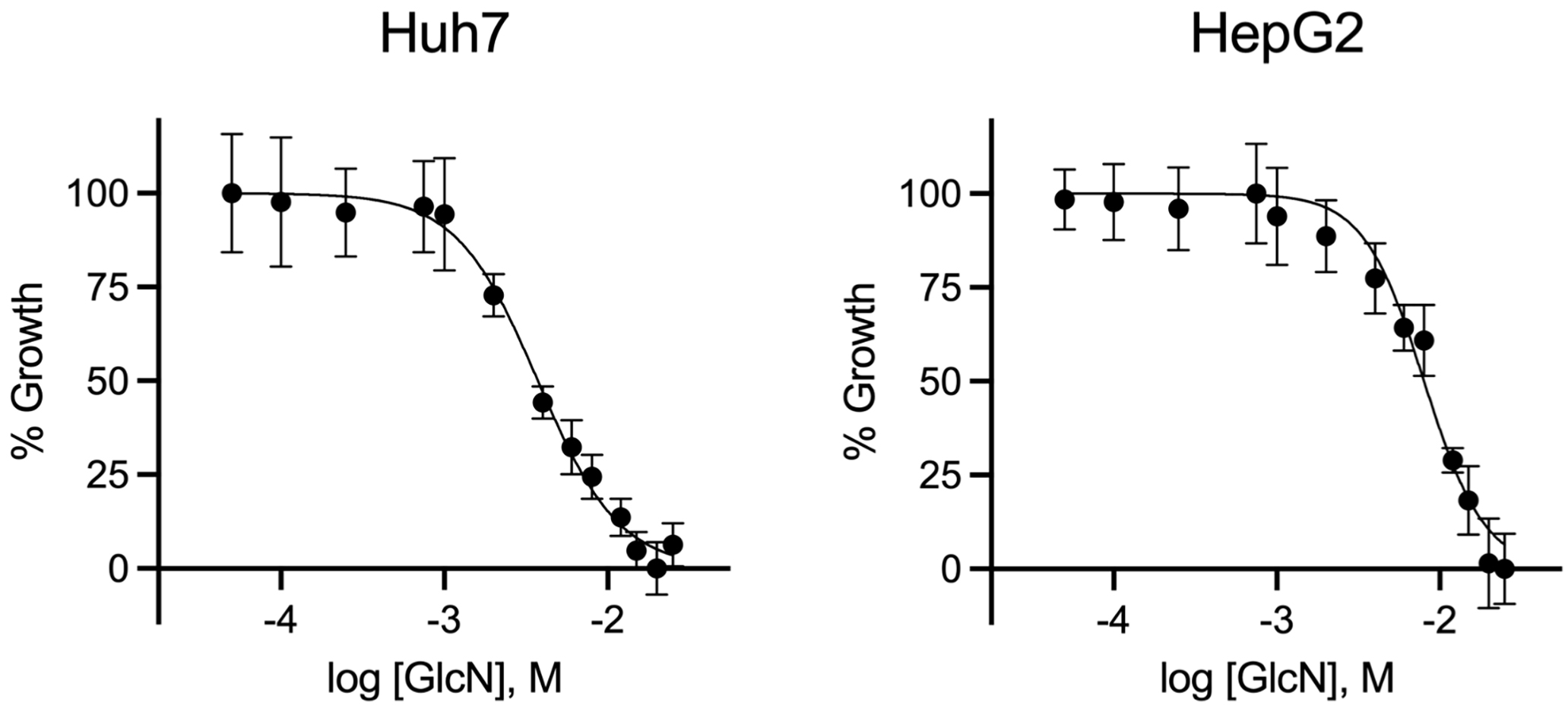

Supplement: FIG S2 [file mbio.03096-22-s0002.jpg]

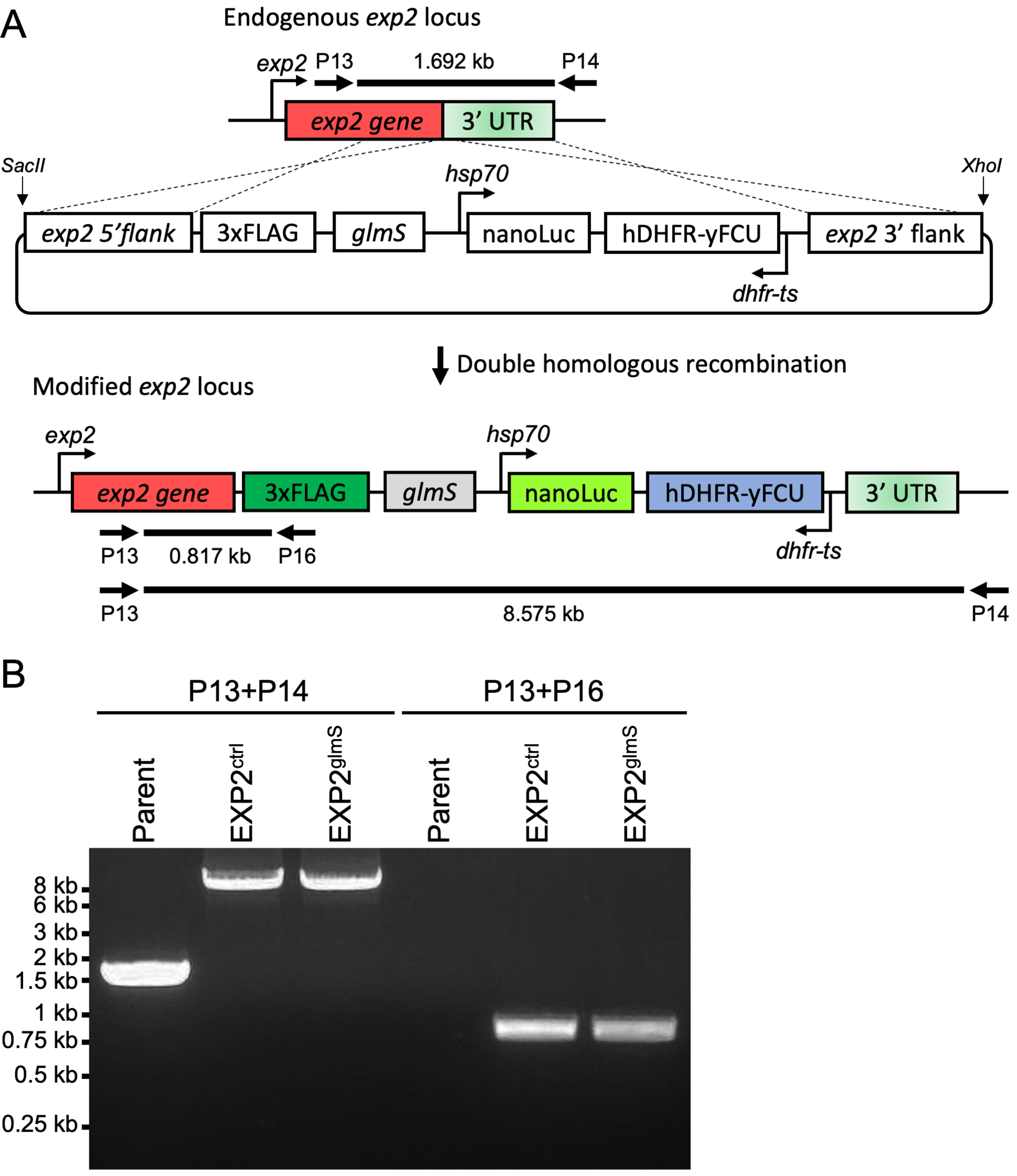

Supplement: FIG S3 [file mbio.03096-22-s0003.jpg]

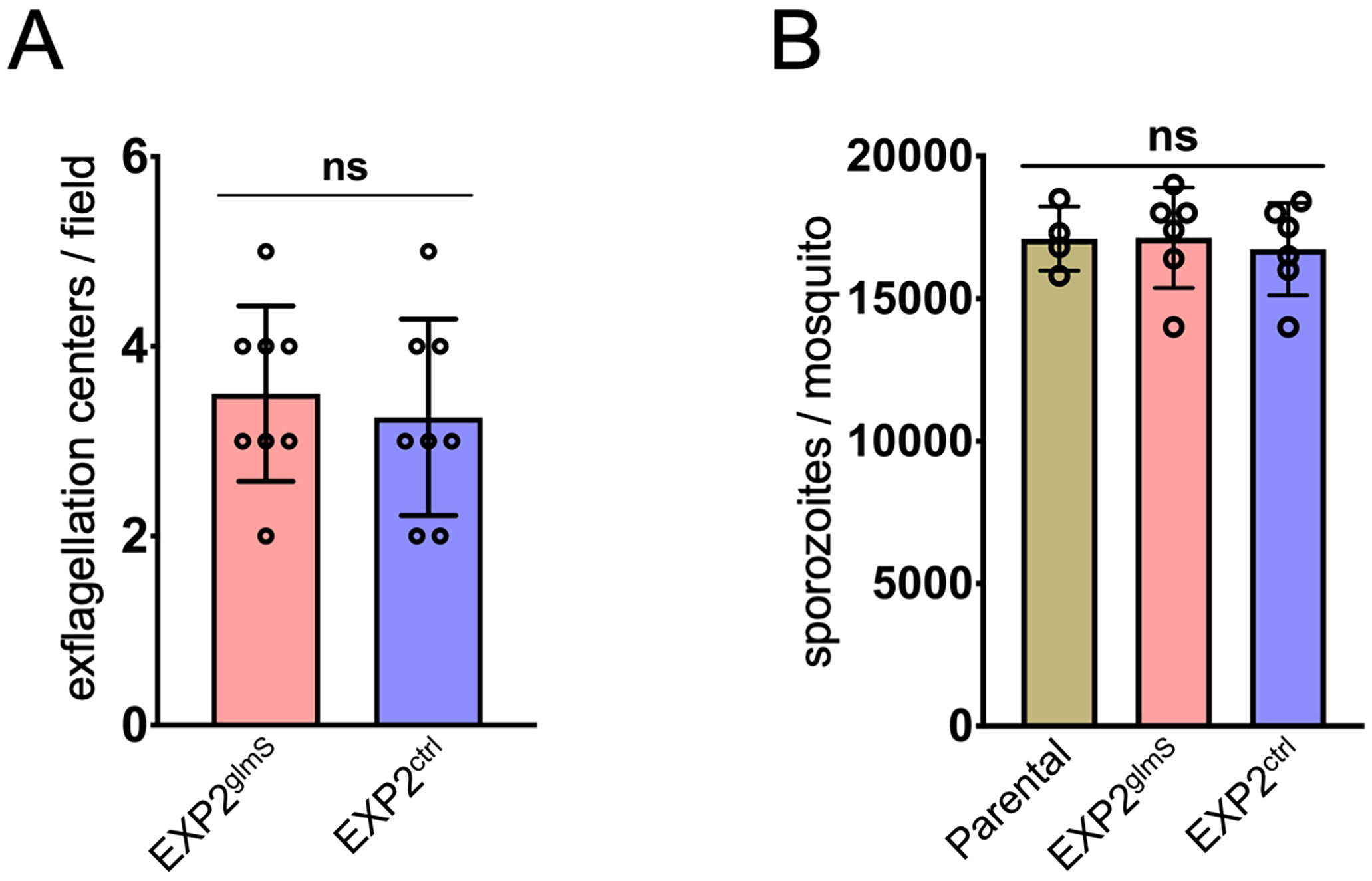

Supplement: FIG S4 [file mbio.03096-22-s0004.jpg]

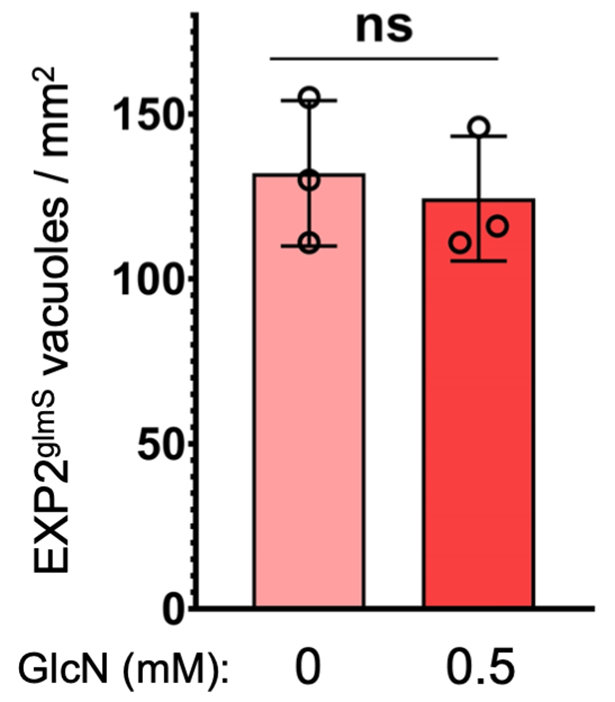

Supplement: FIG S5 [file mbio.03096-22-s0005.jpg]

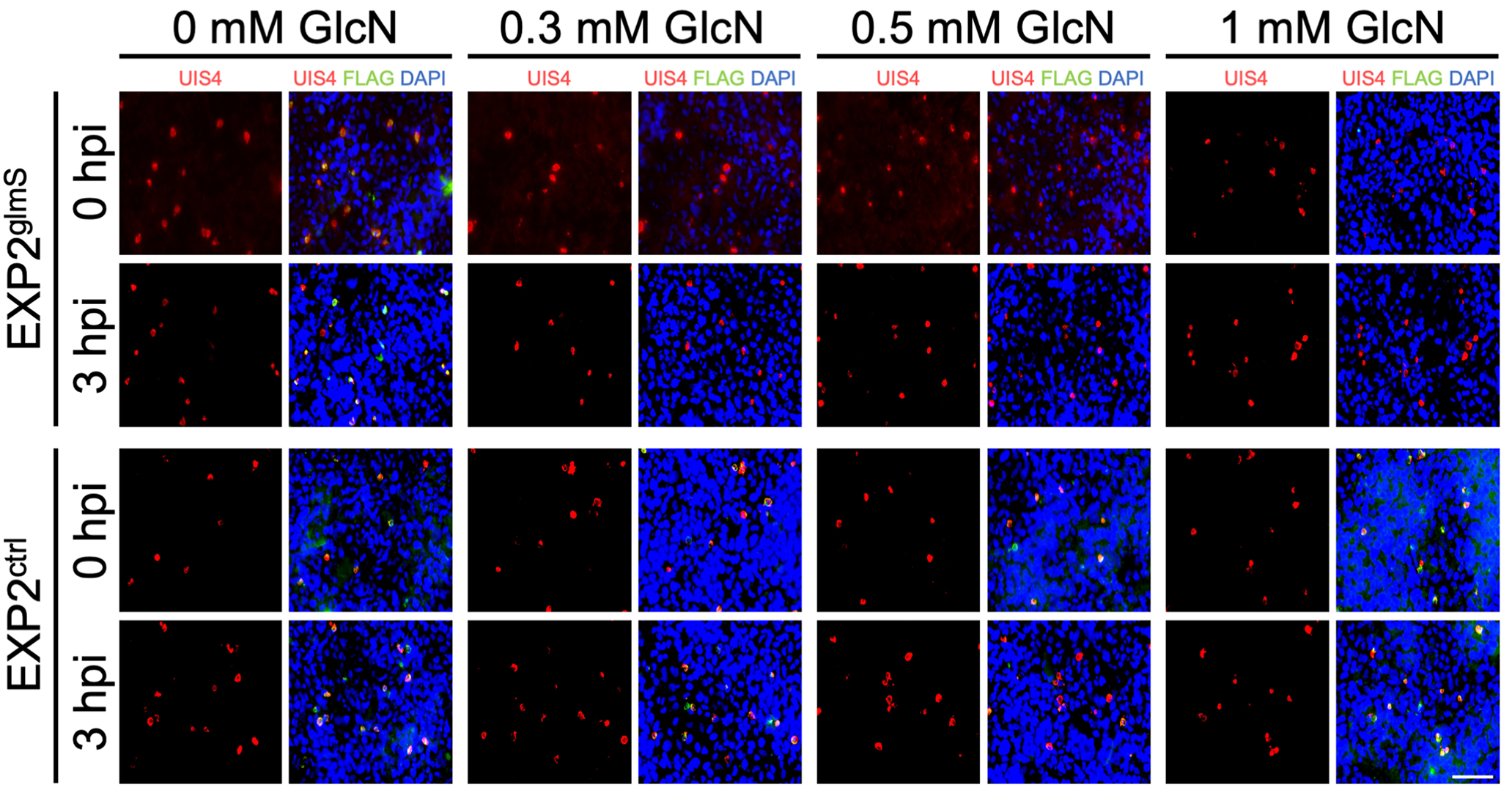

Supplement: FIG S6 [file mbio.03096-22-s0006.jpg]

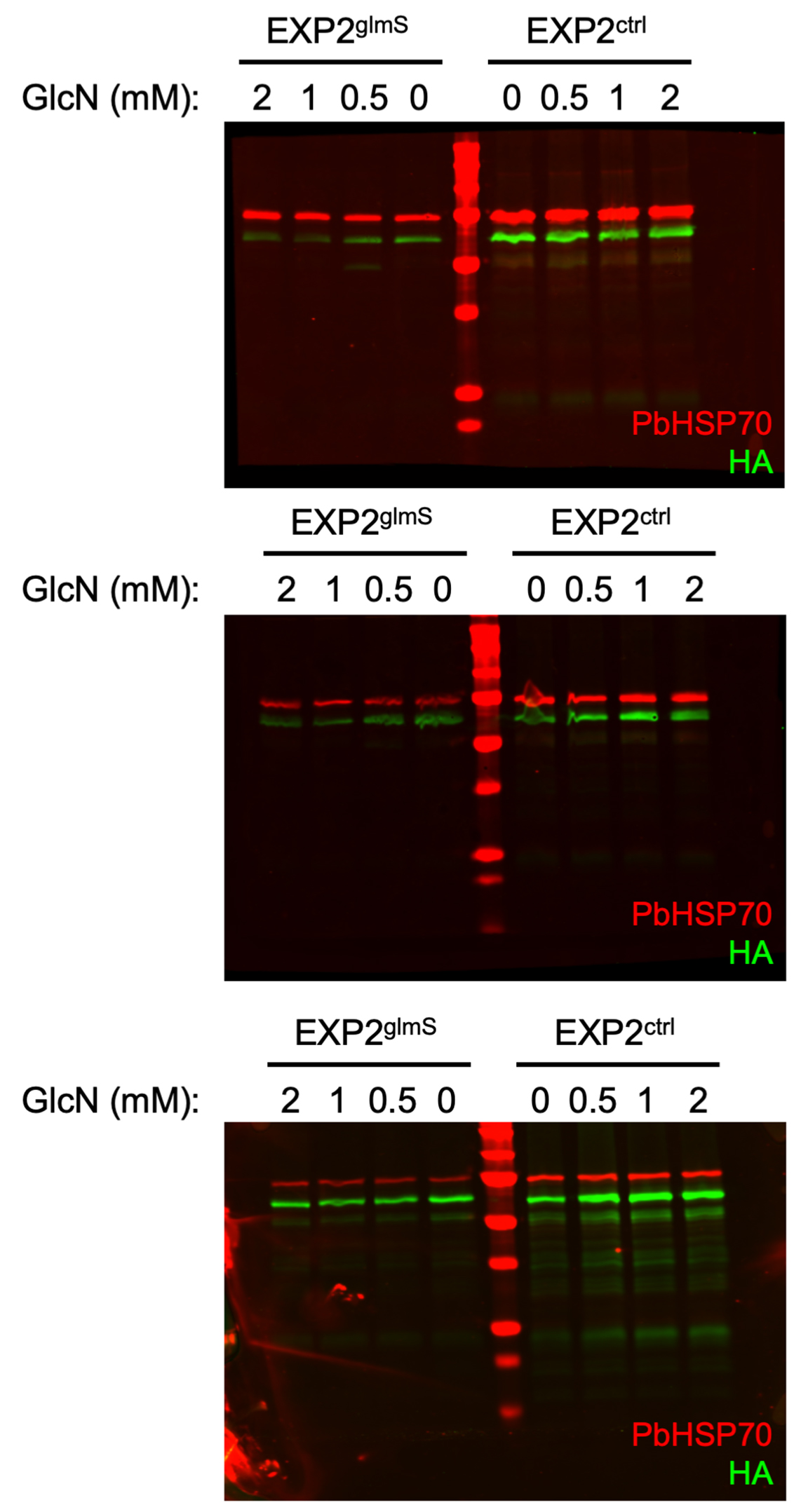

Supplement: FIG S7 [file mbio.03096-22-s0007.jpg]
